# AS-OCT of cyclitic membrane pre and post treatment with rtPA in the anterior chamber

**DOI:** 10.22336/rjo.2022.37

**Published:** 2022

**Authors:** Emma Marín Payá, Marina Aguilar González, Miriam Rahhal Ortuño, Paula Martínez López Corell, Jorge Vila Arteaga, Manuel Díaz Llopis

**Affiliations:** * Department of Ophthalmology, Hospital Universitari i Politecnic La Fe, Valencia, Spain

**Keywords:** intracameral rtPA, cyclitic membrane, pupillary block, AS-OCT

## Abstract

We present the case of a 40-year-old male, who presented to the ophthalmology emergency department with pain and visual loss in his left eye 10 days after an intravitreal injection of a split medication. At the exploration, we found an intense corneal edema in the left eye with endothelial dusting and ciliary hyperemia. LE fundus was impracticable due to anterior chamber opacity. Because of the corneal edema, we performed an anterior segment optical coherence tomography (AS- OCT), visualizing a cyclitic membrane with pupillary block and inflammatory cells in the anterior chamber. There are different treatments to lyse the cyclitic membrane; in this case, we managed the cyclitic membrane with 0,05 ml of intracameral recombinant tissue plasminogen activator (rtPA), a highly potent fibrinolytic protein. We disinfected the eyelids and the conjunctival sac with Povidone Iodine solution, applied topical anesthesia with double anesthetic, and injected 0,05 mL rtPA solution into the anterior chamber using an insulin syringe with a 30-gauge needle.

Intracameral rtPA was prepared under sterile conditions using 50 mg vials of rtPA diluted with 50 mL of sterile water to create a 1 mg/ mL solution. Four hours after rtPA treatment, the cyclitic membrane lysed, obtaining pupillary mydriasis. The AS OCT before and after the treatment with intracameral rtPA was of high utility as it allowed the visualization of the cyclitic membrane and its removal.

## Introduction

Cyclitic membrane is a fibrous proliferation as a result of severe inflammation and ischemia [**1**,**2**]. Fibrinous membrane formation in the anterior chamber may result in reduced visual acuity, photophobia, pupillary block glaucoma, fixed miotic pupil resistant to topical mydriatics, IOL displacement, posterior capsule opacification and loss of ciliary body integrity, which can lead to decreased aqueous production, hypotony, macular edema, and phthisis [**3**-**5**].

There are different treatments to lyse the cyclic membrane. Intracameral injection of recombinant tissue plasminogen activator (rtPA), a highly potent fibrinolytic protein, has been shown to successfully lyse fibrin membranes. Resolution of inflammatory membrane occurred within 24 hours after intracameral rtPA [**4**,**6**].

Reported uncommon complications of intracameral rtPA include corneal edema, band keratopathy, anterior chamber turbidity, hyphaema, and IOL opacification [**6**].

## Case presentation

**Fig. 1:**
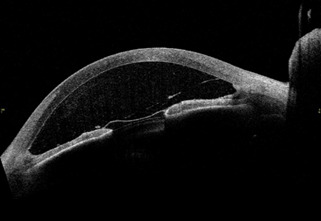
AS-OCT pre-rtPA treatment. Pupillary cyclitic membrane is observed, as well as the inflammation in the anterior chamber and a secondary pupillary block

A 40-year-old male went to the ophthalmology emergency department referring pain and loss of vision in his left eye 10 days after an intravitreal injection of a split medication. Visual acuity (VA) was 1 in the right eye (RE) and hand motion in the left eye (LE). Intraocular pressure was 12 mmHg in RE and 35 mmHg in LE. Biomicroscopic examination highlighted the existence of an intense corneal edema in the left eye with endothelial dusting, ciliary hyperemia, cyclitic membrane with pupillary blockage and inflammatory cells in the anterior chamber (2 +). LE fundus was impracticable due to anterior chamber opacity. Biomicroscopic and ophthalmoscopic examination of the RE was normal. Anterior segment optical coherence tomography (AS- OCT) was carried out, in which the cyclitic membrane together with the inflammation in the anterior chamber was visualized. A secondary pupillary block could also be seen (**Fig. 1**).

The cyclitic membrane was treated with intracameral rtPA after disinfection of the eyelids and the conjunctival sac with Povidone Iodine solution, and topical anesthesia with double anesthetic. 

**Fig. 2:**
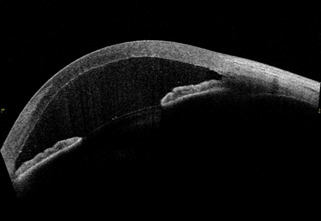
AS-OCT four hours post-rtPA treatment. After 4 hours of administering 0.05 mL of rtPA in the anterior chamber, the cyclitic membrane disappeared, with greater pupillary mydriasis

Intracameral rtPA was prepared under sterile conditions using 50 mg vials of rtPA diluted with 50 mL of sterile water to create a 1 mg/ mL solution. Using an insulin syringe with a 30-gauge needle, 0.05 mL of this solution were injected into the anterior chamber. Four hours after rtPA treatment, a new AS-OCT was performed and lysis of the cyclitic membrane with greater pupillary mydriasis was visualized (**Fig. 2**). 

Thanks to AS-OCT, we managed to photograph the cyclitic membrane before treatment, and the result after applying rtPA. 

## Conclusion

To our knowledge, it is the first published image of a cyclitic membrane pre and post rtPA treatment in AS-OCT.

**Conflict of Interest Statement**

The authors state no conflict of interest. 

**Informed Consent and Human and Animal Rights statement**

Informed consent has been obtained from the patient included in the study.

**Authorization for the use of human subjects**

Ethical approval: The research related to human use complies with all the relevant national regulations, institutional policies, it is in accordance with the tenets of the Helsinki Declaration and has been approved by the review board of Hospital Universitari i Politecnic La Fe, Valencia, Spain. 

**Acknowledgements**

None. 

**Sources of Funding**

None. 

**Disclosures**

None. 
